# Factors Associated with the Choice of Contraceptive Method following an Induced Abortion after Receiving PFPS Counseling among Women Aged 20–49 Years in Hunan Province, China

**DOI:** 10.3390/healthcare11040535

**Published:** 2023-02-10

**Authors:** Chenxi Tong, Yang Luo, Ting Li

**Affiliations:** Xiangya Nursing School, Central South University, Changsha 410013, China

**Keywords:** contraceptive counselling, contraceptive methods, post abortion care, contraception

## Abstract

Background: There is limited research on postabortion family planning (PAFP) services and subsequent contraception in China. The current study aimed to identify women’s contraceptive methods choices and associated factors after receiving PAFP services. Methods: A cross-sectional study used a cluster, stratified and multistage random sample to collect data. All eligible data were analyzed using SPSS 26.0. The chi-square test was used to assess the association between categorical variables. Significant variables (*p* < 0.05) and all potential variables were then included in the binary logistic regression model for analysis. Results: Approximately 84.7% (1043/1231) of participants had received pre-abortion PAFP counselling, and approximately 90% of them chose reliable methods. Farmers or workers (OR = 0.297, 95% CI: 0.130–0.683), family monthly income (3000–4999 RMB, OR = 0.454, 95% CI: 0.212–0.973; ≥5000 RMB, OR = 0.455, 95% CI: 0.228–0.909), reliable advice from services providers before abortion (OR = 0.098, 95% CI: 0.039–0.250), painless surgical abortion (OR = 3.465, 95% CI 1.177–10.201), and postabortion follow-up (OR = 0.543, 95% CI: 0.323–0.914) and were associated with contraception choice after receiving PAFP services. Conclusions: This study emphasizes the importance of pre-abortion PAFP counselling, postabortion follow-up, and increased focus on women who have experienced painless abortion. The study provides direction for PAFP services policymakers, as well as a reference for contraceptive counselling research around the world.

## 1. Introduction

Approximately 121 million unintended pregnancies occur in the world every year, with 61% of them ending in induced abortions (IAs) [1]. Aiming at breaking the cycle of repeated and unintended pregnancy, Ipas, an organization that works with partners around the world to advance reproductive justice by expanding access to abortion and contraception, first proposed the term “postabortion care” (PAC) in its 1991 strategic planning document [2]. PAC consists of five core parts: community and service provider partnerships, counselling, treatment, and reproductive and other health services [2]. The 1994 International Conference on Population and Development (ICPD) recognized PAC as a fundamental strategy, and many countries have committed themselves to protecting women’s reproductive rights [3]. In a Turkish study, contraceptive counselling increased participants’ use of contraceptives by 36.3% and use of modern contraceptive methods by 62.0% [4]; additionally, contraceptive counselling reduced the rate of repeated IAs by approximately 50% in Russia [5]. Furthermore, PAC was found to significantly reduce maternal mortality and associated social costs [6,7].

Based on successful international experiences, China launched a PAC programme in 2011, focusing on family planning and contraceptive services, and issued guidelines for postabortion family planning (PAFP) services [8]. PAFP services in China refer to a holistic service that includes one-on-one PAFP counselling before IA, abortion care, and follow-up after IA [8]. PAFP services have been shown to be associated with reduced unintended pregnancies, repeat IAs, increased women’s relevant reproductive health knowledge, and increased use of long-acting contraceptive methods [9,10,11]. China still has continued to have approximately 10 million abortions per year since 2014 [12], and approximately 50% are repeat IAs [13]. It may relate to the choice of contraceptive method.

Factors associated with choosing contraceptive methods are complex, including family planning policies, individual characteristics, and the availability, affordability, and acceptability of contraceptive methods [14,15]. Some studies have also investigated factors related to contraceptive use among women who have received PAFP/contraceptive counselling. A study in England found that using a contraceptive method after contraceptive counselling in women associated with a history of IA was related to age, IA history, and IA method [16]. Another study in Tanzania found that the content of contraceptive counselling, the doctor’s counselling skills, women’s knowledge of reproduction, and the side effects of contraceptives all had an impact on their uptake of contraceptives [17]; other possible factors include contraceptive decision-making autonomy, contraceptive supplies, and well-trained service providers [18,19].

However, there is limited research on PAFP services and subsequent contraception in China. A review of the literature found that two studies conducted interviews with service stakeholders [20] and service recipients of PAFP service [21] to understand the current development of PAFP service in China. Two cross-sectional studies investigated the relationship between PFPF service and service providers [22], as well as Factors related to women seeking PAFP service [23]. None of these studies involved women’s contraceptive choices after receiving PAFP services. In addition, Hou et al. [24] investigated the continuous use of contraceptive methods among women who attended contraceptive counselling, but their results only for condoms, intrauterine devices (IUDs), and combined oral contraceptives (COCs), while the use of other methods and the associated factors are uncertain, it is also unclear how abortion care and subsequent follow-up relate to contraceptive choice.

Therefore, this study aimed to identify contraceptive method choices after receiving PAFP counselling and factors associated with PAFP services among women aged 20–49 in Hunan Province, China.

## 2. Materials and Methods

### 2.1. Study Design and Participants

The current study was a secondary analysis of the Women’s Health Needs Survey data. The original study was a cross-sectional study conducted in Hunan Province from April to September 2018 by the Hunan Provincial Women’s Federation and the local government (details can also be found in the previously published article [25]). It used a cluster-stratified, multistage random sample to investigate the health needs of women in four age groups (10–19, 20–39, 40–49, and 50–70 years). To provide a diverse and representative sample, first, we chose five cities at random from Hunan’s eastern, western, southern, northern, and central regions. Second, one district representing an urban area and one county representing a rural area were randomly selected from each of the sampled cities. Third, ten communities were chosen at random from the districts and counties that were sampled; the random method used in each step is the random number table method. Finally, all women between the ages of 10 and 70 years in these areas who had lived in Hunan for more than a year and could communicate in Chinese were included in the study; women who chose not to participate or could not communicate effectively were excluded. We selected data from individuals aged 20–49 years in the current study.

**Figure 1:**
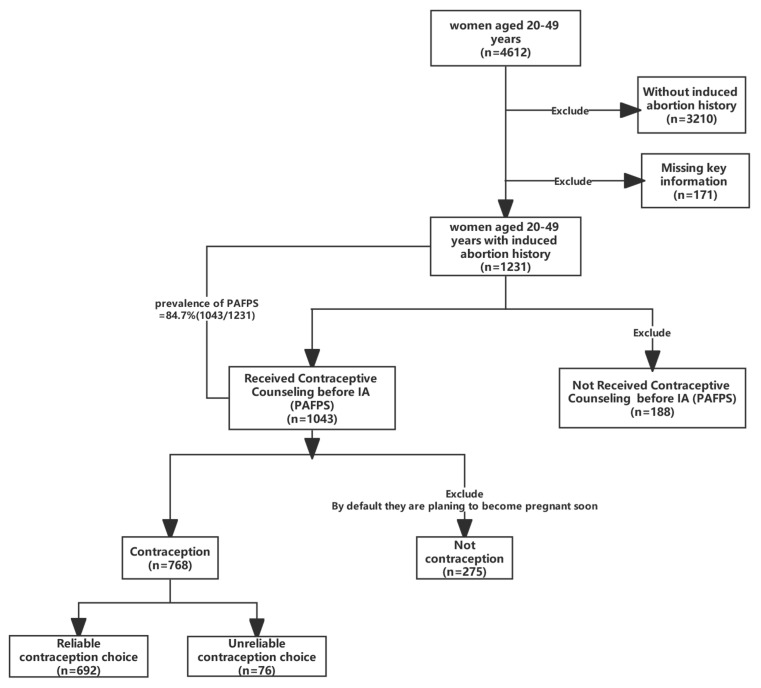
The flow chart of this study.

We excluded women with no history of IA (*n* = 3210) and questionnaires missing key information (*n* = 171) from the total of 4612 women aged 20–49 years, leaving 1231 women with a history of IA. Then, 188 women who had not received PAFP counselling before IA were excluded, leaving 1043 women who received PAFP counselling. Then, to determine the contraceptive method choices after receiving PAFP counselling and associated factors, we excluded those women who did not use contraception; by default, they were considered to have a current plan to become pregnant (*n* = 275). Finally, 768 women were included (Figure 1).

Trained investigators conducted face-to-face questionnaire interviews with women to collect data. The questionnaires were developed after an extensive literature study and evaluated the relevance of all items (Including sociodemographic characteristics, reproductive health history and reproductive health-related knowledge, etc.) by a panel of 10 experts from Central South University, an affiliated hospital, and the Hunan Provincial Women’s Federation.

### 2.2. Variables

#### 2.2.1. Sociodemographic Characteristics

The sociodemographic characteristics consisted of age, residence, occupation, education level, marital status, personal monthly income, family monthly income, and the number of children. Residence was divided into urban and rural areas. Occupation includes unemployed, farmer or worker, professional or administrative staff, staff business or service personnel, and others. We ranked educational levels as low, middle, and high. Low indicated that the participant received 0~9 years of education, middle indicated 10~12 years of education, and high indicated more than 12 years of education. Marital status was classified as married (including married, remarried, and cohabitation) and single (including single, separated, divorced, or widowed). We categorized family monthly income into <3000 RMB (1 RMB ≈ 0.1573$, 30 March 2022), 3000–4999 RMB, and ≥5000 RMB, and the classification of personal monthly income was none, <3000 RMB, 3000–4999 RMB, and ≥5000 RMB. The number of children was classified as 0, 1 and ≥2 according to the number of children currently alive

#### 2.2.2. Contraceptive Methods

The World Health Organization (WHO) categorizes contraceptive methods based on their effectiveness, i.e., the number of women per 100 who used a specific method of contraception but the method failed; the effectiveness is classified as follows: (1) very effective (0–0.9 pregnancies per 100 women), (2) effective (1–9 pregnancies per 100 women), (3) moderately effective (10–19 pregnancies per 100 women), and (4) less effective (20 or more pregnancies per 100 women) [26]. Contraceptive use can be divided into typical use, which refers to all women who use the method, including those who use it inconsistently and incorrectly, and perfect use, which includes only those who use the method both consistently and correctly [27]. Male/female sterilization, IUDs, and implants are defined as “very effective” [26]. They have failure rates of less than 1% for both typical and perfect use [26,27]. COCs are short-acting hormonal methods; they have a failure rate of approximately 7% when typically used but are considered “very effective” when perfectly used [26,27]. Additionally, condoms are also the only contraceptive method that can prevent sexually transmitted infections, and they are widely available; although they are only classified as “effective” when perfectly used [26,28], they are advised as a short-term contraceptive strategy after IA or in combination with other methods to reduce the risk of sexually transmitted infections [29,30]. Emergency contraceptive pills (ECPs) are “very effective” as a remedy after unprotected sex but do not work the next time [26]. ECPs are also not recommended as a continuous contraceptive method because it is uncertain whether ECPs taken every time after sex are as effective as regular, continuous contraceptive methods [26]. Other methods, such as rhythm, withdrawal, spermicides, and diaphragms, are considered “less effective” when typically used [26,27]. The latest PAFP services guidelines [30] and the Consensus of Chinese Experts [29] recommend that women undergo sterilization or use implants, IUDs, COCs, or condoms after IA.

In the current study, contraceptive methods were classified as reliable and unreliable. Participants were classified as using “reliable” contraceptive methods as long as they used any of the following methods: male/female sterilization, implants, IUDs, COCs, and condoms. Otherwise, their methods (including ECPs, rhythm, withdrawal, spermicides, and diaphragms) were classified as “unreliable”.

#### 2.2.3. Factors Associated with PAFP Services

PAFP services include PAFP counselling before IA, IA care and follow-up after IA. In the current study, women who received PAFP counselling prior to IA were then able to answer the next questions.

Factors associated with PAFP counselling included four questions. The first question, “What contraceptive methods did service providers tell you about?” was a multiple-choice question, and the answers were then used to classify participants as having reliable or unreliable methods. The question “How many contraceptive methods did the service provider introduce you to?” included the following responses: “recommended only one method,” “recommended various methods,” and “others”. The next question was, “Were you informed about the side effects of the contraceptive methods?” (yes/no). Finally, the question “How many factors did you know were risk factors for having an induced abortion?” was also a multiple-choice question; each correct option was given a score of one, and an incorrect option was given a score of zero, for a maximum score of 5 points.

IA care included three questions. A history of IA was measured as 1 or ≥2. IA methods included medical abortion, painless surgical abortion, nonpainless surgical abortion, and medical combined with surgical abortion. The painless surgical abortion method indicated that general anaesthesia was used during the abortion procedure, while the nonpainless method indicated that no anaesthesia was used during the abortion procedure. Satisfaction was divided into satisfied, neutral, and dissatisfied according to participants’ experience.

Follow-up after IA, women were asked, “Did you receive one-on-one contraceptive counselling one month after IA?” (yes/no). According to PAFP services guidelines [30], women need to return to the clinic for a one-on-one contraceptive consultation one month after the abortion.

### 2.3. Statistical Analysis

Categorical variables were analysed by descriptive statistics, including frequencies and percentages. The chi-square test was used to compare the differences between participants with reliable and unreliable contraceptive methods. The reported *p* values were two-sided, and a value of *p* < 0.05 indicated statistical significance. Two binary logistic regression models were used to calculate odds ratios (ORs) and 95% confidence intervals (CIs) to analyse the factors associated with reliable contraception. Variables that were significant in the chi-square test were included in model a, and the other potential variables combined with the significant variables in model b were adjusted. Data entry was carried out in Epi-Data version 3.0, and the Statistical Package for Social Sciences (SPSS) v26.0 (IBM, Chicago, IL, USA) was used for the statistical analysis.

## 3. Results

### 3.1. Contraceptive Methods

**Figure 2:**
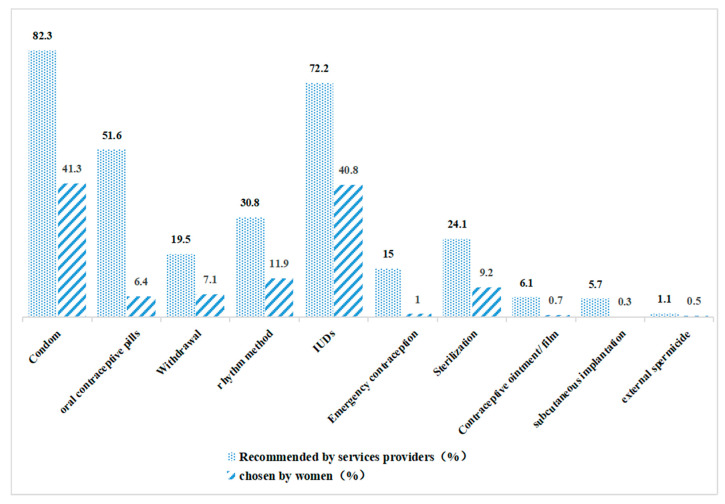
Contraceptive Methods Chosen by Women and Recommended by Services Providers.

A total of 692/768 (90.1%) participants used reliable contraceptive methods (Figure 1). Figure 2 shows the details of contraceptive methods. Service providers recommended condoms, IUD, and COCs to 82.3%, 72.2%, and 51.6% of participants, respectively, but no more than half of participants used any of the three methods, and the use of COCs was particularly poor (41.3%, 40.8%, and 6.4%, respectively). As a long-acting, reversible method with a very high contraceptive rate, implants were rarely recommended by service providers (5.7%) and were rarely ever used (0.7%).

### 3.2. Characteristics of the Participants

Table 1 shows that 59.5% of the 768 women were 40–49 years old, and almost all the participants were married (94.1%). More than half (59.8%) were urban residents, 43.2% had a low educational level, and the most reported occupation was farmer or worker (37.1%). Approximately 53.6% of participants reported a ≥5000 RMB family monthly income, and 47.9% reported a <3000 RMB personal monthly income. A total of 45.8% of the women had fewer than two children.

### 3.3. PAFP Services

Figure 1 illustrates that 84.7% (1043/1231) of women received PAFP counselling prior to abortion. Then, as shown in Table 2, nearly half of all participants had repeated IAs (46.5%). The most common IA method was painless surgical abortion (52.3%), and 459 (53.4%) women were satisfied with their previous IA experience.

Additionally, among the 768 women who received PAFP counselling and were using contraception, almost all participants (774, 96.9%) were recommended to use reliable contraceptive methods, 445 (57.9%) were recommended to use various methods, and 646 (84.1%) were informed of the methods’ side effects. In addition, when participants were asked how much they knew about the potential risk of IA, 249 (32.4%) of them received a score of zero, only 86 (7.0%) received a score of four points, and no one received a score of more than four. (Table 2).

Table 2 also shows that the number of participants who had a one-on-one visit one month after their IA was 437 (56.9%).

### 3.4. Factors Associated with Contraception Choice

According to the chi-square test, women with middle education levels (93.2%) were more likely to use reliable methods than women with low (91.0%) and high education levels (81.4%; *p* < 0.05; Table 1). Farmers and workers (95.4%) were more prone to use reliable methods than women in other occupations (unemployed 86.7%, professional or administrative staff 83.6%, staff business or service personnel 88.5%, others 91.7%, separately; *p* < 0.05; Table 1). Compared with women who used other abortion methods (painless surgical abortion 88.1%, nonpainless surgical abortion 93.2%, and combined medical and surgical abortion 85.7%), women who had a medical abortion (96.2%; *p* < 0.05; Table 2) for the last IA were most likely to use reliable methods. In addition, the proportion of women who had received a one-on-one consultation after their IA was significantly higher than that of women who had not been followed up (92.2% and 87.3%, respectively; *p* < 0.05; Table 2). The proportion of use of reliable methods was also significantly greater among women who were told about reliable methods by service providers (91.3%) than among those who were told about unreliable methods (54.2%; *p* < 0.05; Table 2).

As shown in Table 3, binary logistic regression analysis was used to explore the factors associated with contraceptive methods. The significant variables from the univariate analysis, including the level of education, occupation, method of the last IA, follow-up, and reliable advice, were introduced into the binary logistic regression equation in model a. We found that farmers and workers were 70.3% significantly less likely than unemployed individuals to use unreliable contraceptive methods (OR = 0.297, 95% CI: 0.130–0.678). Women who chose painless surgical abortion for the last IA were more likely to use unreliable contraceptive methods than those who used medical abortion (OR = 3.353, 95% CI: 1.151–9.769). Women who were followed up after IA were 49.4% less likely to use unreliable contraceptive methods than those who were not (OR = 0.506, 95% CI: 0.303–0.846). Women were 89.1% significantly less likely to use unreliable contraceptive methods if they received reliable advice (OR = 0.109, 95% CI: 0.044–0.274).

We further adjusted for all potential variables in model b to control for potential confounding factors, and the variables with statistical significance are presented in Table 3. Similar to the results of model a, occupation as a farmer or worker (OR = 0.297, 95% CI: 0.130–0.678), reliable advice (OR = 0.098, 95% CI: 0.039–0.250) follow-up (OR = 0.543, 95% CI: 0.323–0.914) were recognized to reduce the use of unreliable contraceptive methods, while painless surgical abortion (OR = 3.465, 95% CI 1.177–10.201) was a risk factor for using unreliable methods. Moreover, family monthly income (OR = 0.454, 95% CI: 0.212–0.973; OR = 0.455, 95% CI: 0.228–0.909) was also associated with contraception; the higher the family income was, the lower the possibility of unreliable contraception.

## 4. Discussion

To our knowledge, our study was the first to assess the relationship between contraceptive method choice and the current status of PAFP services in China. Women’s choice of contraceptive methods after receiving PAFP counselling was associated with occupation, family monthly income, advice from service providers before IA, IA method, and follow-up after IA.

Approximately 85% of participants received PAFP counselling in our study, but a previous survey reported that only 57% of service providers in China provided such services [28], a figure significantly lower than ours. This could be related to disparities in medical service resource distribution and different management systems in each region. The prevalence is also higher than 20% in Ghana [31] and 31.2% in northern Tanzania [17]. However, China still lags behind other countries, such as the United States, where 96% of women were found to receive PAFP services [32], and northern Ethiopia, where 98% were found to receive such services [33], indicating that China still needs to improve the rate of counselling. Interviews with service providers and women may help policymakers understand obstacles to contraceptive counselling delivery and establish policies to improve the prevalence of such counselling.

Consistent with previous studies in China [34], we found a relationship between occupation and the use of contraceptive methods. Farmers and workers were more likely than participants with other occupations to use reliable methods in this study. As shown in other studies worldwide, wealthier people are more likely to be able to afford reliable contraceptives [35,36].

In this study, women’s reliable contraceptive use was highly associated with reliable advice from service providers. Other research has also demonstrated that healthcare knowledge affects participants’ perceptions [21,37,38]. The most recommended and used contraceptive methods were condoms and IUDs in our study, but the rhythm method, with a high contraceptive failure rate, was also commonly recommended. In contrast, only 5.7% of providers recommended implants as a reliable contraceptive method. This indicates that the knowledge of contraceptive methods of service providers needs to be strengthened, consistent with previous research in China [20,22,39]. Although the data of “recommendations by service providers” is actually “remembered recommendations by service providers”, the results may be affected by participants’ memories. Due to a lack of relevant knowledge, many PAFP services providers are unable to provide correct and comprehensive contraceptive knowledge [22]; therefore, policymakers should encourage service providers to conduct regular knowledge training or attend academic conferences on reproduction and contraception to stay up to date on the latest developments in the field.

The IA method was found to be related to contraceptive methods. Painless surgical abortions were three times more likely to lead to unreliable contraception than medical abortions. The possible reasons are that painless surgical abortion is convenient and fast, with negligible sensory trauma to women, so the pain of the surgery is quickly forgotten afterwards, making it difficult for them to adhere to a reliable contraceptive method. Furthermore, another study indicated that perceiving the abortion procedure as painless was positively associated with repeated abortion [40]. In contrast, one study [41] showed that long-term safe and reversible postabortion contraception uptake was slightly lower among women treated with medical abortion than surgical abortion because of the convenience of inserting an IUD immediately after the operation. The finding has important implications for our study, and service providers should advise women to use long-acting reversible contraceptive methods that do not require self-control, such as IUDs and implants, immediately after a painless surgical abortion, thereby reducing the future possibility of using unreliable methods of contraception.

Additionally, one-on-one contraceptive counselling one month after IA, also called follow-up, could prevent the choice of unreliable contraception. Moreover, follow-up could provide individualized suggestions to improve contraceptive adherence and reduce contraceptive discontinuation. However, only approximately half of the participants returned to the clinic for a one-on-one consultation one month after the IA. On the one hand, this might be because the service provider cannot provide consultation due to limited appointment availability [22]. On the other hand, women may think that receiving counselling again is unnecessary. Some modern mobile technologies can supplement or replace existing face-to-face follow-up services to improve contraceptive adherence, reduce contraceptive discontinuation, or encourage changing rather than quitting contraceptives when women have side effects. These include text, voice, video, applications, and interactive counselling based on text combined with telephone or video counselling [42,43,44].

The study found that more than half of women were recommended to use COCs, but less than 10% used them, possibly because they were afraid of the side effects. Although our study did not find a relationship between side effects and contraceptive methods, other studies in China found that patients refused to use reliable contraception because they did not feel familiar with those methods and were worried about side effects [21,45]. Complete counselling services during PAFP services should inform people of various contraceptive methods and their side effects to choose their contraceptive methods based on informed consent [8]. A limited choice of contraceptive methods and inadequate information both lead to unreliable contraception [46]. Although most of the participants were told about side effects, nearly half were told only one method in this study. One possibility is that women cannot fully remember all the information provided by service providers due to the recall bias in the questionnaire survey; other possible reasons may be related to the limited time of the consultation service or limited knowledge of the service provider. Service providers can inform patients about contraceptive methods and side effects in easy-to-understand ways, such as through leaflets [47]. Moreover, they can also inform them about reproductive knowledge through this method.

According to the latest Chinese PAFP services guidelines [30], service providers should inform women about abortion-related reproductive health knowledge when providing counselling services. However, our data showed that 32% of participants did not know any potential risks, indicating a significant lack of knowledge. Although no correlation between female reproductive health knowledge and the use of reliable contraceptive methods was found in this study, other studies confirmed it [21,48,49]. As a result, future research can explore how to improve women’s reproductive health knowledge uptake after they have received PAFP services.

Compared to previous findings in China that women’s choice of contraceptive method after participating in contraceptive counselling was related to age, education level, marriage, reproductive history, and abortion history, our study adds new findings and focuses more on the correlation between holistic PAFP services and subsequent contraceptive choice. This study investigated the prevalence of PAFP services based on a community population. Moreover, the study was stratified through random sampling, so our results were representative. Some limitations to note are that the study collects data through questionnaires, participants may have had some recall bias, and women might have refused to answer the questionnaire because topics such as abortion and contraception were too sensitive for them, but our questionnaire was anonymous and had a large sample size, so this bias was avoided to some extent. In addition, since there was no question asking women about their current pregnancy plans, those who did not currently plan to become pregnant but were not using contraception were excluded. Finally, we cannot determine causality because of the nature of cross-sectional studies.

## 5. Conclusions

This study emphasizes the importance of pre-abortion PAFP counselling, postabortion follow-up, and increased focus on women who have experienced painless abortion. The study provides direction for PAFP services policymakers, as well as a reference for contraceptive counselling research around the world.

## Figures and Tables

**Table 1:** Sociodemographic Characteristics of Participants by Efficacy of Chosen Contraceptive (*n* = 768).

Variables	Contraceptive Methods			χ²	*p*-Value
	Total (*n* = 768)	Reliable (*n* = 692)	Unreliable (*n* = 76)		
Age (years)				0.719	0.698
20–29	103 (13.4%)	91 (88.3%)	12 (11.7%)		
30–39	208 (40.5%)	186 (89.4%)	22 (10.6%)		
40–49	457 (59.5%)	415 (90.8%)	42 (9.2%)		
Residence				2.628	0.105
Urban	459 (59.8%)	407 (88.7%)	52 (11.3%)		
Rural	309 (40.2%)	285 (92.2%)	24 (7.8%)		
Education level ^#1^				15.364	<0.001 *
Low	332 (43.2%)	302 (91.0%)	30 (9.0%)		
Middle	296 (38.5%)	276 (93.2%)	20 (6.8%)		
High	140 (18.2%)	114 (81.4%)	26 (18.6%)		
Occupation				17.610	0.001 *
Unemployed	113 (14.7%)	98 (86.7%)	15 (13.3%)		
Famer or worker	285 (37.1%)	272 (95.4%)	13 (4.6%)		
Professional or Administrative staff	134 (17.4%)	112 (83.6%)	22 (16.4%)		
Staff Business or Service personnel	200 (26.0%)	177 (88.5%)	23 (11.5%)		
Others	36 (4.7%)	33 (91.7%)	3 (8.3%)		
Marital status ^#2^				0.054	0.816
Being single	45 (5.9%)	41 (91.9%)	4 (8.9%)		
Being married	723 (94.1%)	651 (90.0%)	72 (10.0%)		
Personal monthly income (RMB) ^#3^				1.478	0.687
None	108 (14.1%)	97 (89.8%)	11 (10.2%)		
<3000	368 (47.9%)	336 (91.3%)	32 (8.7%)		
3000–4999	230 (29.9%)	205 (89.1%)	25 (10.9%)		
≥5000	62 (8.1%)	54 (87.1%)	8 (12.9)		
Family monthly income (RMB) ^#3^				1.830	0.401
<3000	151 (19.7%)	132 (87.4%)	19 (12.6%)		
3000–4999	205 (26.7%)	188 (91.7%)	17 (8.3%)		
≥5000	412 (53.6%)	372 (90.3%)	40 (9.7%)		
Number of children				1.464	0.481
0	13 (1.7%)	13 (100%)	0		
1	403 (52.5%)	362 (89.8%)	41 (10.2%)		
≥2	352 (45.8%)	317 (90.1%)	35 (9.9%)		

^#1^: Low means received 0–9 years education; Middle means received 10–12 years education; High means received more than 12 years of education; ^#2^: Being married including married, remarried, and cohabitation; Being single including single, separated, divorced, or widowed; ^#3^: 1 RMB ≈ 0.1573$, 30 March 2022; * *p* < 0.05.

**Table 2:** Factors Associated with Induced Abortion and PAFP services by Efficacy of Chosen Contraceptive (*n* = 768).

Variables	Contraceptive Methods			χ²	*p*-Value
	Total (*n* = 768)	Reliable (*n* = 692)	Unreliable (*n* = 76)		
What contraceptive methods did the service providers tell you about?				35.884	<0.001 *
Unreliable methods	24 (3.1%)	13 (54.2%)	11 (45.8%)		
Reliable methods	744 (96.9%)	679 (91.3%)	65 (8.7%)		
How many contraceptive methods did the service provider introduce you to?				2.251	0.324
Recommend only one method	302 (39.3%)	271 (89.7%)	31 (10.3%)		
Recommend various methods	445 (57.9%)	404 (90.8%)	41 (9.2%)		
Others	21 (2.7%)	17 (81.0%)	4 (19.0%)		
Were you informed about the side effects of the contraceptive methods?				1.685	0.194
No	122 (15.9%)	106 (86.9%)	16 (13.1%)		
Yes	646 (84.1%)	586 (90.7%)	60 (9.3%)		
How many factors did you know were risk factors for having an induced abortion?					
Points				5.646	0.227
0 ^#4^	249 (32.4%)	232 (93.2%)	17 (6.8%)		
1	149 (19.4%)	132 (88.6%)	17 (11.4%)		
2	184 (24.0%)	167 (90.8%)	17 (9.2%)		
3	124 (16.1%)	109 (87.9%)	15 (12.1%)		
4	62 (8.1%)	52 (83.9%)	10 (16.1%)		
5	0				
Induced abortion history				0.614	0.685
1	411 (53.5%)	372 (90.5%)	39 (9.5%)		
≥2	357 (46.5%)	320 (89.6%)	37 (10.4)		
What procedure did you undergo with your most recent induced abortion?				9.990	0.019 *
Medical abortion	105 (13.7%)	101 (96.2%)	4 (3.8%)		
Painless surgical abortion	402 (52.3%)	354 (88.1%)	48 (11.9%)		
Non-painless surgical abortion	177 (23.0%)	165 (93.2%)	12 (6.8%)		
Medical combined with surgical abortion	84 (10.9%)	72 (85.7%)	12 (14.3%)		
Were you satisfied with your most recent abortion services?				1.214	0.545
Satisfied	459 (59.8%)	412 (89.8%)	47 (10.2%)		
Neutral	274 (35.7%)	250 (91.2%)	24 (8.8%)		
Dissatisfied	35 (4.6%)	30 (85.7%)	5 (14.3%)		
Did you receive one-on-one contraceptive counselling one month after your IA?				5.089	0.024 *
No	331 (43.1%)	289 (87.3%)	42 (12.7%)		
Yes	437 (56.9%)	403 (92.2%)	34 (7.8%)		

**^#^**^4^: Based on the answers chosen by the participants, each correct option was credited with a score of one and an incorrect option with a score of zero and a maximum score of 5 points; * *p* < 0.05.

**Table 3:** Binary logistic Regression Analysis of Potential Factors Associated with Contraceptive Method Choice (*n* = 768).

	Model a ^#5^			Model b ^#6^		
Variables	*p*	ORs	95% CIs	*p*	ORs	95% CIs
Occupation						
Unemployed	–	–	–	–	–	–
Farmer or Worker	0.004 *	0.297	0.130–0.678	0.004 *	0.297	0.130–0.678
Professional or Administrative staff	0.946	1.030	0.437–2.426	0.946	1.030	0.437–2.426
Staff business or Service personnel	0.679	0.854	0.404–1.805	0.679	0.854	0.404–1.805
Others	0.158	0.350	0.081–1.506	0.158	0.350	0.081–1.506
Family monthly income (RMB)						
<3000				–	–	–
3000–4999				0.042 *	0.454	0.212–0.973
≥5000				0.026 *	0.455	0.228–0.909
Unreliable methods	–	–	–	–	–	–
Reliable methods	<0.001 *	0.109	0.044–0.274	0.000 *	0.098	0.039–0.250
Medical abortion	–	–	–	–	–	–
Painless surgical abortion	0.027 *	3.353	1.151–9.769	0.024 *	3.465	1.177–10.201
Non-painless surgical abortion	0.328	1.811	0.551–5.949	0.303	1.879	0.566–6.246
Medical and surgical abortion	0.080	2.987	0.879–10.145	0.058	3.290	0.961–11.261
No	–	–	–	–	–	–
Yes	0.009 *	0.506	0.303–0.846	0.021 *	0.543	0.323–0.914

^#^^5^: model a included the variables of the level of education, occupation, the methods of the last IA, follow-up, and reliable advice; ^#6^: mode b included the variables of the level of education, occupation, the methods of the last IA, follow-up, and reliable advice, and age, residence, marital578status, income, and number of children and IA history; * *p* < 0.05.

## Data Availability

Not applicable.
